# Supplementary material for: An approach to social flexibility: Congruency effects during spontaneous word-by-word interaction
Source: PLoS One. 2020 Jun 24;15(6):e0235083. doi: 10.1371/journal.pone.0235083 (PMC7313956; doi:10.1371/journal.pone.0235083)
Supplement: S1 Table — *significance at p < 0.05 **significance at p < 0.001. (DOCX) [file pone.0235083.s001.docx]

| **Table 1**: Pearson correlation between all measures | | | | | | | | | | | |
| --- | --- | --- | --- | --- | --- | --- | --- | --- | --- | --- | --- |
|  |  | **TTRT** |  | **CE** |  | **Number-**  **Letter** |  | **GoNogo** |  | **Stop-**  **Signal** |  |
| **TTRT** | *r* | - |  |  |  |  |  |  |  |  |  |
|  | *p* | - |  |  |  |  |  |  |  |  |  |
| **Congruency Cost** | *r* | -0.103 |  | - |  |  |  |  |  |  |  |
|  | *p* | 0.434 |  | - |  |  |  |  |  |  |  |
| **Number-Letter** | *r* | -0.022 |  | -0.151 |  | - |  |  |  |  |  |
|  | *p* | 0.868 |  | 0.249 |  | - |  |  |  |  |  |
| **GoNogo** | *r* | 0.053 |  | 0.130 |  | 0.183 |  | - |  |  |  |
|  | *p* | 0.690 |  | 0.324 |  | 0.161 |  | - |  |  |  |
| **Stop-Signal**^[[1]](#footnote-1)^ | *r* | 0.059 |  | 0.318 | * | 0.057 |  | 0.430 | ** | - |  |
|  | *p* | 0.653 |  | 0.013 |  | 0.665 |  | < .001 |  | - |  |
| *significance at *p* < 0.05  **significance at *p* < 0.001 | | | | | | | | | | | |

1. Correlation between CE and non-corrected SSRTs also reached significance with *r*= 0.301, *p*= 0.019. [↑](#footnote-ref-1)
