# Supplementary material for: An approach to social flexibility: Congruency effects during spontaneous word-by-word interaction
Source: PLoS One. 2020 Jun 24;15(6):e0235083. doi: 10.1371/journal.pone.0235083 (PMC7313956; doi:10.1371/journal.pone.0235083)
Supplement: S1 Data — (DOCX) [file pone.0235083.s002.docx]

**Supplementary Material S1**

We rated the subjective relation between context word (CW), target word 1 (TW1), and target word 2 (TW2). English translation of the original stimuli in brackets.

Reported are means (M) and standard error over the mean (SEM). The relation between TW1 and TW2 were rated by 20 participants, all other words are rated by 10 participants.

**Condition: congruent** (same target word)

| **context word** | **target word** | **CW – TW**  n = 10 | |
| --- | --- | --- | --- |
|  |  | M (SEM) | |
| Garten (garden) | Bart (beard) | 0,20 | (0,13) |
| Café (café) | Tasche (bag) | 0,50 | (0,20) |
| Einkaufszentrum (shopping center) | Rohr (pipe) | 0,10 | (0,10) |
| Spielzeuggeschäft (toyshop) | Wecker (alarm clock) | 0,80 | (0,39) |
| Hotel (hotel) | Sparschwein (piggybank) | 1,20 | (0,39) |
| Drogerie (drugstore) | Palme (palm tree) | 0,40 | (0,31) |
| Flohmarkt (flea market) | Käse (cheese) | 0,30 | (0,21) |
| Demonstration (demonstration) | Pfanne (pan) | 0,20 | (0,13) |
| Vorstellungsgespräch (job interview) | Bonbon (bonbon) | 0,40 | (0,22) |
| Urlaub (vacation) | Erdnuss (peanut) | 0,30 | (0,21) |
| Sauna (sauna) | Arzt (doctor) | 0,50 | (0,17) |
| Klassenfahrt (class trip) | Feile (file (tool)) | 0,40 | (0,22) |
| Hochzeit (wedding) | Apfel (apple) | 0,30 | (0,21) |
| Spieleabend (board games night) | Teller (plate) | 0,60 | (0,34) |
| Lehrerzimmer (teachers‘ room) | Socke (sock) | 0,10 | (0,10) |
| Tanzkurs (dancing course) | Schleife (bowknot) | 0,90 | (0,28) |
| Dschungel (jungle) | Nase (nose) | 0,00 | (0,00) |
| Beerdigung (funeral) | Haus (house) | 0,90 | (0,28) |
| Berge (mountains) | Katze (cat) | 0,00 | (0,00) |
| Krieg (war) | Teppich (carpet) | 0,10 | (0,10) |
| Fahrstuhl (elevator) | Buch (book) | 0,30 | (0,21) |
| Chorprobe (choir practice) | Schere (scissors) | 0,10 | (0,10) |
| MC Donalds | Pinsel (paintbrush) | 0,00 | (0,00) |
| Wartezimmer (waiting room) | Besteck (cutlery) | 0,10 | (0,10) |
| Videoverleih (video rental) | Gans (goose) | 0,10 | (0,10) |
| Burg (castle) | Aal (eel) | 0,20 | (0,20) |
| Sportplatz (athletics field) | Drucker (printer) | 0,00 | (0,00) |
| Schlittschuhbahn (ice rink) | Kamin (chimney) | 0,50 | (0,31) |
| Umzug (relocation (person)) | Kuh (cow) | 0,00 | (0,00) |
| Kirche (church) | Bürste (brush) | 0,10 | (0,10) |

**Condition:** incongruent (different but related target words)

| **context word** | **target word 1** | **target word 2** | **TW1-TW2**  n = 20 | | **CW-TW1**  n = 10 | | **CW-TW2**  n = 10 | |
| --- | --- | --- | --- | --- | --- | --- | --- | --- |
|  |  |  | M (SEM) | | M (SEM) | | M (SEM) | |
| Park (park) | Lehrer (teacher) | Schüler (pupil) | 3,75 | (0,12) | 0,50 | (0,22) | 1,10 | (0,28) |
| Zirkus (circus) | Korb (basket) | Pilz (mushroom) | 2,05 | (0,31) | 0,40 | (0,22) | 0,00 | (0,00) |
| Disko (disco) | Wohnung (apartment) | Küche (kitchen) | 3,30 | (0,16) | 0,50 | (0,22) | 0,40 | (0,22) |
| Bus (bus) | Hut (hat) | Kopf (head) | 3,60 | (0,21) | 0,30 | (0,15) | 0,30 | (0,21) |
| Geburtstagsparty (bithday party) | Fischer (fisherman) | Hering (herring) | 3,30 | (0,23) | 0,10 | (0,10) | 0,50 | (0,22) |
| Zuhause (home) | Hals (neck) | Bauch (belly) | 2,35 | (0,26) | 0,10 | (0,10) | 0,60 | (0,27) |
| Büro (office) | Floß (raft) | Boot (boat) | 3,00 | (0,30) | 0,00 | (0,00) | 0,20 | (0,13) |
| Bahnhof (train station) | Torwart (goal keeper) | Spieler (player) | 3,60 | (0,13) | 0,00 | (0,00) | 0,70 | (0,30) |
| Bibliothek (library) | Brot (bread) | Ei (egg) | 2,60 | (0,17) | 0,20 | (0,20) | 0,20 | (0,13) |
| Restaurant (restaurant) | Fahrrad (bicycle) | Pedal (pedal) | 3,75 | (0,12) | 0,50 | (0,17) | 0,10 | (0,10) |
| Kino (cinema) | Fuß (foot) | Schuh (shoe) | 3,80 | (0,12) | 0,70 | (0,26) | 0,50 | (0,22) |
| Fitnessstudio (gym) | Sonne (sun) | Wolke (cloud) | 3,30 | (0,18) | 0,50 | (0,22) | 0,00 | (0,00) |
| Museum (museum) | Gabel (fork) | Nudeln (pasta) | 3,15 | (0,21) | 0,10 | (0,10) | 0,20 | (0,13) |
| Ikea (ikea) | Stein (stone) | Weg (path) | 2,80 | (0,22) | 0,60 | (0,22) | 0,90 | (0,23) |
| Skipiste (ski slope) | Stuhl (chair) | Tisch (table) | 3,45 | (0,18) | 0,30 | (0,15) | 0,50 | (0,22) |
| Küche (kitchen) | Koffer (suitcase) | Rucksack (backpack) | 3,10 | (0,22) | 0,20 | (0,13) | 0,20 | (0,13) |
| Zoo (zoo) | Kissen (pillow) | Sofa (sofa) | 3,60 | (0,13) | 0,10 | (0,10) | 0,10 | (0,10) |
| Theater (theatre) | Tasse (cup) | Flasche (bottle) | 2,30 | (0,27) | 0,40 | (0,40) | 0,40 | (0,22) |
| Picknick (picnic) | Fußmatte (doormat) | Haustür (front door) | 3,50 | (0,22) | 0,30 | (0,21) | 0,60 | (0,22) |
| Fußballspiel | Hirsch (stag) | Bär (bear) | 2,35 | (0,23) | 0,00 | (0,00) | 0,10 | (0,10) |
| Tierheim (animal shelter) | Zwerg (dwarf) | Riese (giant) | 2,95 | (0,26) | 0,30 | (0,21) | 0,40 | (0,22) |
| Labor (laboratory) | Krabbe (crab) | Muschel (mussel) | 3,25 | (0,20) | 0,50 | (0,27) | 0,30 | (0,15) |
| Polizeistation (police station) | Blume (flower) | Wiese (pasture) | 3,55 | (0,18) | 0,40 | (0,22) | 0,40 | (0,22) |
| Schwimmbad (public swimming pool) | Kühlschrank (fidge) | Toaster (toaster) | 2,35 | (0,23) | 0,20 | (0,13) | 0,20 | (0,13) |
| Gefängnis (prison) | Kuchen (cake) | Kaffee (coffee) | 3,60 | (0,15) | 0,50 | (0,22) | 0,70 | (0,30) |
| Hafen (harbor) | Auge (eye) | Gehirn (brain) | 2,90 | (0,19) | 0,10 | (0,10) | 0,20 | (0,13) |
| Familienfeier (family celebration) | Eule (owl) | Taube (pigeon) | 2,70 | (0,26) | 0,10 | (0,10) | 0,70 | (0,26) |
| Turnhalle (gymnasium) | Kerze (candle) | Torte (gateau) | 2,90 | (0,26) | 0,00 | (0,00) | 0,40 | (0,22) |
| Raumschiff (spacecraft) | Jacke (jacket) | Hose (pants) | 3,30 | (0,15) | 0,30 | (0,21) | 0,10 | (0,10) |
| Bushaltestelle (bus stop) | Schnitzel (escalope) | Messer (knife) | 2,55 | (0,29) | 0,20 | (0,20) | 0,50 | (0,27) |
